# Effects of microRNA-305 knockdown on brain gene expression associated with division of labor in honey bee colonies (*Apis mellifera*)

**DOI:** 10.1242/jeb.246785

**Published:** 2024-04-30

**Authors:** Sarai H. Stuart, Amy C. Cash Ahmed, Laura Kilikevicius, Gene E. Robinson

**Affiliations:** ^1^ Program in Ecology, Evolution, and Conservation Biology, University of Illinois Urbana-Champaign, Urbana, IL 61801, USA; ^2^ Carl R. Woese Institute for Genomic Biology, University of Illinois Urbana-Champaign, Urbana, IL 61801, USA; ^3^ Neuroscience Program, University of Illinois Urbana-Champaign, Urbana, IL 61801, USA; ^4^ Department of Entomology, University of Illinois Urbana-Champaign, Urbana, IL 61801, USA

**Keywords:** miRNA, Transcriptomics, Behavioral maturation, Transcription factor, Sociality, Gene regulation

## Abstract

Division of labor in honey bee colonies is based on the behavioral maturation of adult workers that involves a transition from working in the hive to foraging. This behavioral maturation is associated with distinct task-related transcriptomic profiles in the brain and abdominal fat body that are related to multiple regulatory factors including juvenile hormone (JH) and queen mandibular pheromone (QMP). A prominent physiological feature associated with behavioral maturation is a loss of abdominal lipid mass as bees transition to foraging. We used transcriptomic and physiological analyses to study whether microRNAs (miRNAs) are involved in the regulation of division of labor. We first identified two miRNAs that showed patterns of expression associated with behavioral maturation, *ame-miR-305-5p* and *ame-miR-375-3p*. We then downregulated the expression of these two miRNAs with sequence-specific antagomirs. Neither *ame-miR-305-5p* nor *ame-miR-375-3p* knockdown in the abdomen affected abdominal lipid mass on their own. Similarly, knockdown of *ame-miR-305-5p* in combination with JH or QMP also did not affect lipid mass. By contrast, *ame-miR-305-5p* knockdown in the abdomen caused substantial changes in gene expression in the brain. Brain gene expression changes included genes encoding transcription factors previously implicated in behavioral maturation. The results of these functional genomic experiments extend previous correlative associations of microRNAs with honey bee division of labor and point to specific roles for *ame-miR-305-5p*.

## Teaser

**Summary:** Knockdown of microRNA-305 in abdominal fat body of adult honey bee workers influences division of labor-related brain gene expression but not division of labor-related changes in abdominal lipid.

## INTRODUCTION

Small RNAs, including microRNAs (miRNAs), small interfering RNAs and Piwi-interacting RNAs, play a role in many biological phenotypes and are now widely recognized as important regulators of gene expression. miRNAs have been identified in the western honey bee (*Apis mellifera*), and some have been suggested to be involved in division of labor (Behura and Whitfield, 2010; Greenberg et al., 2012; Liu et al., 2012). We examined the role of specific miRNAs in the regulation of gene expression and related physiological changes associated with division of labor in honey bees.

Honey bee colonies exhibit an age-related division of labor among workers that is based on a pattern of individual behavioral maturation (Johnson, 2023; Robinson, 1992). Adults spend the first 2–3 weeks working in the hive engaged in tasks such as brood care (nursing) before shifting to tasks outside, spending the remainder of their lives foraging for nectar and pollen and/or defending the hive. Honey bees perform some tasks consistently over an extended period of time, such as nursing and foraging (Schulz et al., 1998). This extreme behavioral specialization is thought to be essential to the success of social insects (Oster and Wilson, 1978). Mechanistically, it requires regulatory mechanisms to stabilize and extend behavioral states.

Behavioral maturation in honey bees is regulated by a suite of molecular and physiological factors that affect gene expression in the brain, abdominal fat body and many other organs (Ament et al., 2011; Whitfield et al., 2003). Some, such as juvenile hormone (JH) (Robinson, 1992; Schulz et al., 2002; Sullivan et al., 2000; Whitfield et al., 2006) and queen mandibular pheromone (QMP) (Grozinger et al., 2003; Pankiw et al., 1998), exert causal effects to accelerate or delay behavioral maturation, respectively.

Adult honey bee workers lose approximately 50% of their abdominal lipid mass around the time they transition from working in the hive to foraging outside, and this loss is maintained for the rest of their lives (Ament et al., 2011; Toth and Robinson, 2005). Lipid loss helps regulate behavioral maturation; pharmacological inhibition of lipid synthesis delays the onset of foraging (Toth et al., 2005). Other animals have also evolved the ability to dynamically respond to their environment with changes in body mass and composition (e.g. Hahn and Denlinger, 2011; Pulido, 2007; Rousseau et al., 2003), but how long-term changes are stabilized while individuals maintain biological activity is not well understood.

miRNAs are small noncoding RNA repressors that stabilize gene expression post-transcriptionally (Chakraborty et al., 2020; Hausser and Zavolan, 2014; Lucas et al., 2015a). They have emerged as important regulators of physiological processes in both invertebrates (Bryant et al., 2010; Lucas and Raikhel, 2013; Lucas et al., 2015b) and vertebrates (Cao et al., 2012; Chang and Mendell, 2007; Liang et al., 2007). Mature miRNA sequences contain binding sites that are 6–8 base pairs long and bind to target mRNAs (Doench and Sharp, 2004; Hausser and Zavolan, 2014; Lewis et al., 2003). Such a short active region predicts that a single miRNA may hybridize with and repress multiple cognate target genes (Coyle, 2001; Enright et al., 2003; Issler, 2015).

We hypothesized that miRNAs regulate behavior-related lipid loss in honey bees. To address this hypothesis, we used a combination of bioinformatic analyses and molecular screens. Based on these results, we selected several miRNAs for further interrogation, especially *ame-miR-305-5p*. We then developed *in vivo* antagomir (miRNA inhibitor) (Krützfeldt et al., 2005) treatments, administered them to bees and performed abdominal lipid mass quantification. We additionally tested whether JH and QMP, factors that have opposite effects on lipid level, interact with *ame-miR-305-5p*.

We also hypothesized that *ame-miR-305-5p* regulates gene expression related to honey bee division of labor in the brain and fat body. To address this hypothesis, we again administered antagomir to honey bees as above and performed whole transcriptome sequencing and differential gene expression analysis to explore the effects of fat body *ame-miR-305-5p* knockdown on fat body and brain gene expression across the genome. For the testing of both hypotheses, we used antagomir constructs composed of oligonucleotides that are engineered to repress the expression of endogenous miRNAs (Krützfeldt et al., 2005).

Studies in mammals (Fernández-Hernando et al., 2011; Lynn, 2009), *Drosophila melanogaster* and other fly species (Ling et al., 2017; Teleman and Cohen, 2006; Xu et al., 2003) have shown that miRNA activity is required to maintain constant lipid metabolism and energy homeostasis. It has also been demonstrated that miRNAs affect JH synthesis (Qu et al., 2018), and may therefore contribute to the role of JH in behavioral maturation. But it is unknown whether miRNAs also contribute to long-term changes in lipid level that are a part of organismal life history.

Several studies have examined miRNA expression differences between nurses and foragers, either in brain or whole head analyses (Behura and Whitfield, 2010; Greenberg et al., 2012; Liu et al., 2012). However, causal effects of miRNAs on aspects of division of labor have been minimally studied (Liu et al., 2017).

## MATERIALS AND METHODS

### Bees

Adult worker honey bees were reared at the University of Illinois Urbana-Champaign Bee Research Facility according to standard beekeeping practices. All bees were a mixture of subspecies of *Apis mellifera* Linnaeus 1758, primarily *A. m. ligustica* Spinola 1806.

Nurses and foragers were identified and collected according to established methods (Robinson, 1992; Toth and Robinson, 2005; Whitfield et al., 2003) and were sampled from colonies headed by naturally mated queens. Bees were collected and immediately frozen in liquid nitrogen to preserve RNA integrity and expression levels. Collections were from two different, unrelated colonies.

For all experiments involving antagomir treatment, 1-day-old adult workers were obtained by collecting frames of honeycomb with capped pupae and placing them in an incubator at 34°C and 50% relative humidity until emergence as adult bees. Frames were sourced from colonies headed by queens each inseminated with a single drone (Laidlaw, 1987) to reduce intra-colony genetic variation.

### Bioinformatic prediction of miRNAs associated with division of labor

Two miRNA target prediction programs, RNAhybrid (Rehmsmeier et al., 2004) and PITA (Kertesz et al., 2007), were used to identify putative interactions between known honey bee miRNAs (miRBase r22) (Griffiths-Jones et al., 2006) and genes associated with JH and insulin/insulin-like signaling pathways, which are also known to be involved in division of labor (Ament et al., 2011). Sequences for 3′ untranslated regions of JH/IIS genes were obtained from the most recent build of the honey bee genome, HAv3.1 (Wallberg et al., 2019). Each program was run independently with recommended settings, and then outputs from each were overlapped. These initial results were compared with published honey bee miRNA data sets (Greenberg et al., 2012; Kapheim et al., 2020; Liu et al., 2012) and literature on other organisms (Foronda et al., 2014; Lynn, 2009; Osugi et al., 2015; Poy et al., 2009; Teleman and Cohen, 2006; Ueda et al., 2018). Both conserved and honey-bee-specific miRNAs were retained as candidate miRNAs for breadth. Our *in silico* analysis produced seven candidate miRNAs that were further screened *in vivo*.

### miRNA quantification

Fat body dissections first separated frozen abdomens from whole bodies on dry ice before placement of all abdominal body parts in RNAlater-ICE (Ambion, Thermo Fisher Scientific, Waltham, MA, USA) overnight at −20°C. After 16 h, internal organs were removed (on wet ice) to leave just the fat body attached to abdominal cuticle. Matched whole brains were dissected following similar methods. Frozen heads were separated from remaining bodies and the frons (frontal cuticle) was removed to expose the brain before placement in RNAlater-ICE overnight. Hypopharyngeal glands, muscle and other head components, including the suboesophageal ganglion, were removed.

Total RNA extraction, cDNA synthesis and qPCR were performed according to standard methods (Bryant et al., 2010). A synthetic version of the *Caenorhabditis elegans* miRNA *cel-miR-39-3p* (Qiagen, Hilden, Germany) was added at the tissue homogenization step as a control for miRNA cDNA synthesis and for normalization. All primer sequences can be found in Table S1. qPCR measurements were performed in triplicate and expression was normalized to *cel-miR-39-3p* using the ΔΔ*C*_T_ method (Schmittgen and Livak, 2008).

### Antagomirs

Antagomir constructs (antisense oligonucleotide miRNA inhibitors) (Krützfeldt et al., 2005) were obtained from Dharmacon (now Horizon Discovery, Waterbeach, UK) using the custom single-stranded RNA synthesis module to create specific modifications for stability and permeability. Modifications included a phosphorothioate backbone instead of the typical phosphodiester backbone for stability, an OCH_3_ on the 2′ end of the base instead of the typical OH group, and a 3′ cholesterol group (Bryant et al., 2010). Construct sequences can be found in Table S1.

Antagomirs were designed to target five of the seven candidate miRNAs identified as possibly associated with division of labor based on bioinformatic analysis and fat body expression pattern in nurses versus foragers: *ame-miR-210-3p*, *ame-miR-305-5p*, *ame-miR-375-3p*, *ame-miR-6056-5p* and *ame-miR-9873-3p*. Primers for each miRNA can be found in Table S1.

### Antagomir treatment

One-day-old adult bees were marked on the dorsal surface of the thorax with Testor's enamel and returned to a colony to develop in a standardized and typical hive environment. Paint-marked bees were sampled when they were 3 days old and located on honeycomb frames containing larvae to standardize sampling methods and increase the likelihood of sampling nurse bees. This age was chosen so that downstream changes in lipid mass and gene expression could be measured when bees were 5 days old, prior to natural behavioral shifts that occur during the behavioral maturation process (Toth and Robinson, 2005; Whitfield et al., 2003).

Antagomir treatment was administered orally. This method has been used successfully to knockdown miRNA expression *in vivo* (Cristino et al., 2014; Liu et al., 2017), although the mechanism by which the antagomirs influence specific tissues is unknown. To stimulate feeding, bees were caged for 60 min without food. They were then lightly anesthetized on wet ice before being individually fed 1000 pmol of antagomir suspended in 6 μl of 30% sucrose solution. Control treatments consisted of 6 μl of 30% sucrose solution only. Following treatment, bees were individually caged to prevent bee-to-bee sharing of antagomir treatment via trophallaxis. Individual cages were provisioned *ad libitum* with 30% sucrose solution to standardize diet and prevent any confounding effects from plant products found in pollen and honey. Bees were then placed in an incubator at 34°C and 50% relative humidity for 48 h, and mortality was measured each day. They were then collected in liquid nitrogen for measurement of miRNA expression (described above), JH/IIS-associated mRNA expression (described below) and lipid mass (described below). Following a similar timeline, experiments using pharmacological treatments reduced lipid mass in 4 days (Toth et al., 2005), meaning that it should be possible to observe changes in miRNA expression and lipid mass at this time point.

### JH/IIS-associated gene expression

We used the same dissection and total RNA extraction methods described for miRNA RT-qPCR and the same total RNA for both miRNA and mRNA RT-qPCR. cDNA was synthesized using M-MuLV Reverse Transcriptase (NEB, Ispwich, MA, USA) and RNase Inhibitor, Human Placenta (NEB). An exogenous RNA (*Root Cap Protein 1* from *Arabidopsis thaliana*) was added at the cDNA step to assess cDNA synthesis efficiency. mRNA expression was measured for four genes involved in JH/IIS: *insulin-like peptide receptor* (*InR-1*) (Ament et al., 2008), *Krüppel homolog 1* (*Kr-h1*) (Grozinger et al., 2003), *ultraspiracle* (*USP*) (Ament et al., 2012a) and *vitellogenin* (*Vg*) (Pinto et al., 2000). Primer sequences can be found in Table S1. qPCR measurements were performed in triplicate and expression was normalized to the geometric mean of the expression of endogenous genes *Rp49*, *S8* and *GapDH*.

### Abdominal lipid mass quantification

Abdominal fat body was dissected using the same methods as for RT-qPCR but with 100% ethanol instead of RNAlater-ICE. Because both RT-qPCR and lipid mass quantification require destructive sampling, different bees were used for each analysis; however, bees used for both analyses were treated at the same time as part of the same trial and were from the same colony. Following dissection, lipids were extracted using previously published methods (Ament et al., 2011; Toth et al., 2005). In brief, individual samples were homogenized in 2:1 chloroform:methanol, allowed to extract overnight, filtered through glass wool, evaporated down and suspended in 2 ml of fresh 2:1 chloroform:methanol. To quantify lipid mass, 100 µl of extracted sample was evaporated, 200 μl sulfuric acid was added and the sample was boiled in a hot water bath for 10 min. Two milliliters of vanillin-phosphoric acid reagent was added to each sample, vortexed and allowed to react for 15 min. Absorbance measurements were read in duplicate at 535 nm, and the mass of individual samples was calculated relative to a cholesterol based standard curve.

### Antagomir and juvenile hormone analog (JHA) treatment

Two by two factorial design experiments were conducted to determine the combined effects of JHA and *ame-miR-305-5p*. Antag-305 treatments were performed according to methods above for antag-305 and previously established protocols for the JHA methoprene (Sullivan et al., 2000). On day 1, bees were treated with JHA and on day 3, antag-305. Each trial consisted of four treatment groups: group 1: antag-305+JHA (experimental); group 2: JHA only (JHA control); group 3: antag-305 only (antagomir control); and group 4: no treatment control (baseline). JHA is frequently used in place of JH for its increased chemical stability and stronger effects (Sullivan et al., 2000; Whitfield et al., 2006), and like JH, induces precocious behavioral maturation. Acetone was used as treatment vehicle for JHA and has shown no effect on foraging behavior (Sullivan et al., 2000). Using the same chemical modifications as antag-305, we designed a missense control antagomir (antag-missense) with the sequence 5′-GGGCAAUCGCUCAUGGUCUCUA-3′.

On day 1, 1-day-old adult bees were individually topically treated on the dorsal abdomen with either 4 µl of 200 µg JHA (Central Life Sciences, Schaumburg, IL, USA) suspended in acetone, or 4 µl of acetone alone. Treated bees were placed in plexiglass cages of 55 bees of the same treatment group to avoid treatment transfer. There were 4 cages per group, each provisioned *ad libitum* with honey, 30% sucrose solution and pollen paste (45% ground pollen, 45% honey and 10% water). Cages were then placed in an incubator at 34°C and 50% relative humidity.

On day 3, JHA- and acetone-treated bees were fed either antag-305 or antag-missense control as described above to create the four different treatment groups. Following treatment, bees were individually caged to prevent the oral sharing of treatment and provisioned *ad libitum* with 30% sucrose solution, then placed back in the incubator.

On day 5, 48 h after antagomir treatment, one half of the bees was collected in liquid nitrogen for miRNA expression and lipid analysis to mirror the timeline for expression and lipid analyses above.

On day 8, the remaining bees were collected in liquid nitrogen to test for long-lasting effects on miRNA expression and lipid levels. JHA–antagomir treatments were performed on a total of three unrelated colonies.

### Antagomir and queen mandibular pheromone (QMP) treatment

Oral antag-305 treatments and chronic QMP treatments were performed according to previously established methods (Ament et al., 2011; Grozinger et al., 2003). Bees were treated with fresh QMP daily, beginning on day 1, throughout the experiment, and antag-305 treatment occurred on day 3 as described above. Each trial consisted of four treatment groups: group 1: antag-305+QMP (experimental); group 2: QMP only (QMP control); group 3: antag-305 only (antagomir control); and group 4: no treatment control (baseline). QMP is regularly used to mimic the presence of a queen bee (Ament et al., 2011; Grozinger et al., 2003; Pankiw et al., 1998). Trials lasted for a total of 5 days based on previous experiments that show QMP effects decrease as bees age (Vergoz et al., 2009). Previous work has also shown that QMP treatment causes the largest effects on gene expression 2 to 3 days after exposure (Grozinger et al., 2003) and increases lipid stores after 5 days (Ament et al., 2011). These results suggested that we would be able to detect differences between groups with this experimental timeline.

On day 1, 1-day-old adult bees were placed in plexiglass cages in groups of 55. Half of the cages received 0.1 queen equivalent of synthetic QMP (TempQueen, Intko Supply, Vancouver, BC, Canada) in isopropanol, and the other half received 90% isopropanol and water. A total of 10 µl of each solution was placed on a glass slipcover, evaporated and placed inside the cages. Cages were provisioned *ad libitum* with pollen paste (45% pollen, 45% honey, 10% water), 30% sucrose solution and honey, and held in an incubator at 34°C and 50% relative humidity. This is a standard method for exposing bees to QMP (Pankiw et al., 1998).

On day 3, QMP- or isopropanol-treated bees were individually fed antagomir constructs as above to create the four different treatment groups. To maintain chronic QMP treatment after bees were placed in individual cages, we created 0.5 g spheres of queen candy (confectioner's sugar and corn syrup), 5 µl of 0.05 queen equivalent or isopropanol control were evaporated on the queen candy, and then the queen candy spheres were pressed onto ventilation holes, treatment side inwards, so that bees could access the treatment for continuous exposure. Cages were provisioned with water only (with queen candy as a source of food) and treatments were replaced with fresh QMP every 24 h.

On day 5, bees were collected in liquid nitrogen for miRNA expression and lipid mass analysis. QMP–antagomir treatments were performed on a total of three colonies, unrelated to each other or the colonies used for the JHA experiment.

### Statistical analyses for RT-qPCR and lipid mass measurements

Plots were generated in the software environment R v1.3.1093. Outliers were removed if they were less than or greater than 1.5× the interquartile range and sample size was below *n*=13 for a treatment group. For analyses that included multiple colonies at *n*=9 bees per group per colony, we used linear mixed models that model variation in group-level intercepts (Gelman and Hill, 2007) to account for the high relatedness of honey bees from the same colony and differences across treatment groups. Log-linear mixed models were run with restricted maximum likelihood (REML) using the lme4 package in R (Bates et al., 2015) to estimate the fixed effect of experimental treatment on natural-log transformed responses (i.e. relative gene expression and lipid mass) with colony of origin as a random effect. Data were plotted as the estimated means.

### Transcriptome sequencing

Effects of antag-305 versus antag-missense treatment on gene expression were studied in multiple trials performed over the course of two field seasons (May–October) with a different source colony for each trial. Antagomir-treated bees were first processed for fat body and miRNA RT-qPCR analysis (*n*=five colonies, one colony=one trial) to confirm the knockdown effect. RNA-sequencing (RNA-seq) was performed at the Roy J. Carver Biotechnology Center at the University of Illinois Urbana-Champaign using the same fat body and brain RNA that was used for RT-qPCR. Matched fat body and brain tissue samples from three colonies were sequenced including at least one colony from each of the two field seasons that trials were performed. Sequencing libraries were prepared with Kapa Hyper Stranded mRNA library kit (Roche, Basel, Switzerland), pooled, quantitated by qPCR and sequenced on one S4 lane for 151 cycles from both ends of the fragments on a NovaSeq 6000 (Illumina, San Diego, CA, USA).

### Bioinformatic analyses

Adapters were trimmed and FASTQ reads were aligned to the most recent build of the honey bee genome, HAv3.1 (Wallberg et al., 2019) using the default settings in STAR v2.7.6a (Dobin et al., 2013). Aligned reads were then counted using ‘featureCounts’ (Liao et al., 2014) in the Subread v2.0.0 package (Liao et al., 2013). Count data were imported into R v1.3.1093 for quality checks and analysis with the package edgeR v.3.24.3 (Robinson et al., 2010). Individual samples were screened for viral contamination from common honey bee viruses, including Deformed wing virus (Lanzi et al., 2006). Because these viruses affect brain gene expression, libraries with a percent viral load >0.05% (determined to be a useful diagnostic threshold) were removed from further analysis (Traniello et al., 2020): four fat body samples and two brain samples (Table S2). All samples were found to belong to the antag-missense treatment group. No transcripts were removed for lowly expressed counts, defined as genes that had fewer than one read per million in at least 30 samples (the smallest group) for fat body and 32 samples for brain; there was a total of 8844 and 9118 genes, respectively, for analysis. Trimmed mean of M-values (TMM) normalization was followed by a generalized linear model with treatment group (antag-305 versus antag-ms) as a categorical predictor of gene expression. Differentially expressed genes (DEGs) were determined with edgeR's quasi-likelihood test functions, and DEGs were subjected to a Benjamini–Hochberg correction for multiple tests (Benjamini and Hochberg, 1995) with a false discovery rate (FDR) of <0.05. Raw and processed RNA-seq reads were uploaded to NCBI GEO under GSE200602. Volcano plots were generated using the Enhanced Volcano package v1.14.0 (https://bioconductor.org/packages/EnhancedVolcano).

We conducted gene list overlap analyses to explore whether the DEGs detected in this study include genes previously found to be associated with honey bee division of labor. Gene list overlap analyses were conducted with hypergeometric tests (Traniello et al., 2020); we first tested for enrichment of transcription factors (TFs) within our brain DEG list by comparing all DEGs with a list of known TFs in the honey bee that was obtained from the Regulator Database (http://www.bioinformatics.org/regulator/page.php) (Wang and Nishida, 2015).

For Gene Ontology (GO) enrichment analysis, a one-to-one reciprocal best hit BLAST v2.8.0 (Camacho et al., 2009) was performed to convert honey bee genes to their *D. melanogaster* orthologs. The lists of converted genes, identified as either shared universe (all the shared orthologs that exist between the two species), antag-305 upregulated brain DEGs or antag-305 downregulated brain DEGs, were then submitted to the GOrilla database for GO enrichment analysis and REVIGO for visualization in semantic space (Eden et al., 2009; Hong et al., 2014; Supek et al., 2011). Out of 6341 recognized gene terms (fly orthologs), 6007 were associated with a GO term. GO analysis was not performed on the abdominal fat body DEGs owing to the small number of DEGs.

The list of brain DEGs from our experiment was compared with lists of brain DEGs from previous nurse versus forager comparisons (Alaux et al., 2009; Khamis et al., 2015) using gene overlap comparisons with hypergeometric tests as above. To obtain the lists of nurse-upregulated or forager-upregulated genes, genes from two previous independent studies (Alaux et al., 2009; Khamis et al., 2015) were used if directionally concordant on both lists (Traniello et al., 2020).

## RESULTS

### miRNAs are predicted to regulate JH/IIS-associated gene expression

Bioinformatic analysis with prediction software RNAhybrid resulted in 75 miRNA–protein coding gene interactions with 60 unique miRNAs (*P*<0.05; Table S3). Using the same input sequences, the program PITA predicted 327 miRNA–gene interactions with a minimum free energy less than −10, resulting in 159 unique miRNAs (Table S3). There were 44 unique miRNAs that were predicted to regulate JH/IIS by both programs.

In some cases, the same miRNA was predicted to regulate the same gene by both programs, such as *ame-miR-305-5p* and its putative target, *insulin-like peptide receptor* (*InR-1*). In other cases, a miRNA was predicted to regulate the same gene by both programs plus additional genes by just one of the programs (Table S3). After comparing the initial set of 44 miRNAs predicted by both programs with known functions in the honey bee or other species, we selected seven candidate miRNAs (Table 1). We identified the predicted target gene(s) and in which endocrine signaling system the gene was predicted to be involved. These seven included some miRNAs with the potential for a conserved role in regulating JH/IIS, such as *ame-miR-210-3p*, *ame-miR-305-5p* and *ame-miR-375-3p*, and some that are currently thought to be present only in the honey bee, such as *ame-miR-6043-3p*, *ame-miR-6056-5p* and *ame-miR-9873-3p* (Table 1)*.* We also included one miRNA, *ame-miR-278-3p*, that was differentially expressed in the brain in nurses compared with foragers in a separate study (Y. Ben-Shahar, personal communication). This miRNA was also predicted to regulate the genes *InR-1* and *alpha-2A adrenergic receptor* by the program PITA, but none by RNAhybrid (Table S3).

**Table 1.**
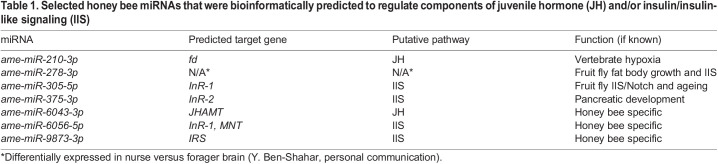
Selected honey bee miRNAs that were bioinformatically predicted to regulate components of juvenile hormone (JH) and/or insulin/insulin-like signaling (IIS)

### miRNAs differed in expression between nurses versus foragers

**Fig. 1.**
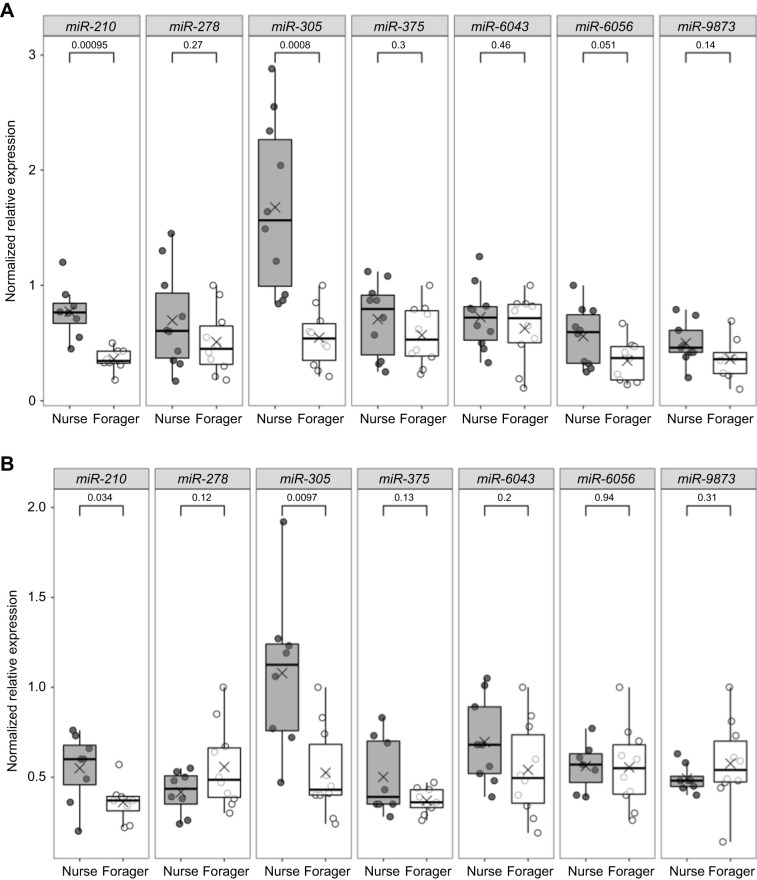
**miRNA expression differed between nurses and foragers.** Nurses and foragers were collected from typical colonies and miRNA expression level measured in the fat body (A) and brain (B) for a subset of candidate miRNAs. Data represented by box plots, Welch's *t*-test, outliers removed, *n*=10 nurse bees and *n*=10 forager bees for fat body, *n*=9 nurse bees and *n*=10 forager bees for brain, from two colonies. Numbers above brackets indicate *P*-values.

Two of the seven candidate miRNAs were significantly differentially expressed in the fat body of nurses and foragers: *ame-miR-210-3p* and *ame-miR-305-5p* (*miR-210* and *miR-305*, respectively) (*P*<0.05, Welch's *t*-test, *n*=7–10 total bees per group, from two colonies; Fig. 1A). Both showed greater than 2-fold abundance in nurses relative to foragers. None of the other miRNAs were differentially expressed in the fat body, including the three honey-bee-specific miRNAs tested.

In the brain, the same two miRNAs, *miR-210* and *miR-305*, were differentially expressed in the same direction and relative magnitude as in the fat body (*P*<0.05; Fig. 1B). Similar to the results for fat body, no other miRNAs in the brain showed significant differences in expression.

### Antagomir treatment reduces miRNA expression

**Fig. 2.**
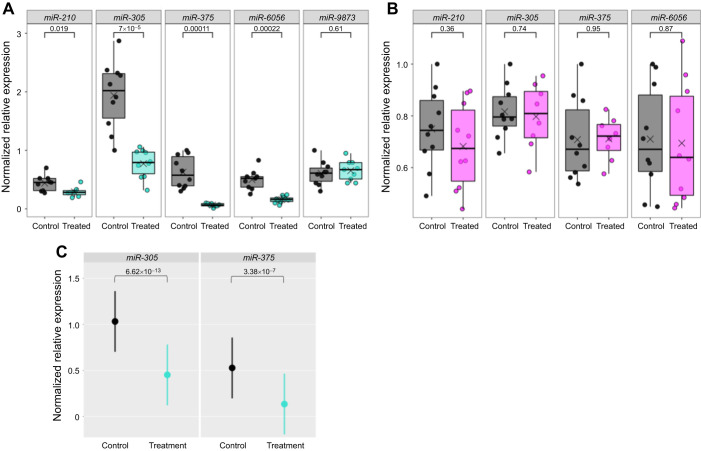
**Oral antagomir (antag) treatment with antag-210, antag-305, antag-375, antag-6056 and antag-9873 decreased expression of miRNAs *ame-miR-210-3p*, *ame-miR-305-5p*, *ame-miR-375-3p* and *ame-miR-6056-5p*, but not *ame-miR-9873-3p*, in the fat body but had no effect in the brain, 48 h after treatment.** (A) miRNA abundance in the fat body for five miRNAs. Sample sizes for each miRNA: miR-210, *n*=7 treated bees and *n*=9 control bees; miR-305, *n*=10 treated bees and *n*=10 control bees; miR-375, *n*=9 treated bees and *n*=10 control bees; miR-6056, *n*=10 treated bees and *n*=9 control bees; miR-9873, *n*=9 treated bees and *n*=10 control bees, all bees were from one colony. (B) miRNA abundance in brain for the four miRNAs that were knocked down in fat body. Sample sizes for each miRNA: miR-210, *n*=10 treated bees and *n*=10 control bees; miR-305, *n*=8 treated bees and *n*=10 control bees; miR-375, *n*=8 treated bees and *n*=10 control bees; miR-6056, *n*=10 treated bees and *n*=10 control bees, all bees were from one colony. Data represented by box plots, Welch's *t*-test, outliers removed. Numbers above brackets indicate *P*-values. (C) Antag-305 and antag-375 treatment consistently and repeatedly reduced *ame-miR-305-5p* (miR-305) and *ame-miR-375-3p* (miR-375) abundance in the fat body, respectively. Linear mixed-effect regression model, colony as a random effect, d.f.=171 for each miRNA, sample sizes for each miRNA: miR-305, *n*=42 treated bees and *n*=41 control bees and miR-375, *n*=42 treated bees and *n*=42 control bees, bees were sourced from four colonies. Circles represent the means and bars represent 95% confidence intervals.

We designed and administered five antagomirs, each designed to target one of five miRNAs. These five were selected from our initial candidate pool of seven miRNAs based on patterns of expression between nurses and foragers: *miR-210*, *miR-305*, *miR-375*, *miR-6056* and *miR-9873*. Fat body miRNA abundance was significantly reduced in four out of the five miRNAs 48 h after treatment (*P*<0.05; Fig. 2A). Measurement of miRNA abundance in the matched brains for these four showed no significant differences (Fig. 2B).

We repeated antag-305 and antag-375 treatments and fat body miRNA expression analysis for bees from three additional unrelated colonies over the course of two field seasons. Mortality for all experiments was <1%. Results showed that antagomir treatment consistently caused a knockdown in miRNA expression for both *miR-305* and *miR-375* (*P*<10^−6^, linear mixed-effect regression, *n*=41–42 bees total per group, from four colonies) and reduced abundance by approximately 50% (Fig. 2C). This knockdown efficiency is consistent with other antagomir studies in the honey bee (Cristino et al., 2014; Liu et al., 2017).

### Antag-305 reduced *Kr-h1* gene expression in the fat body

**Fig. 3.**
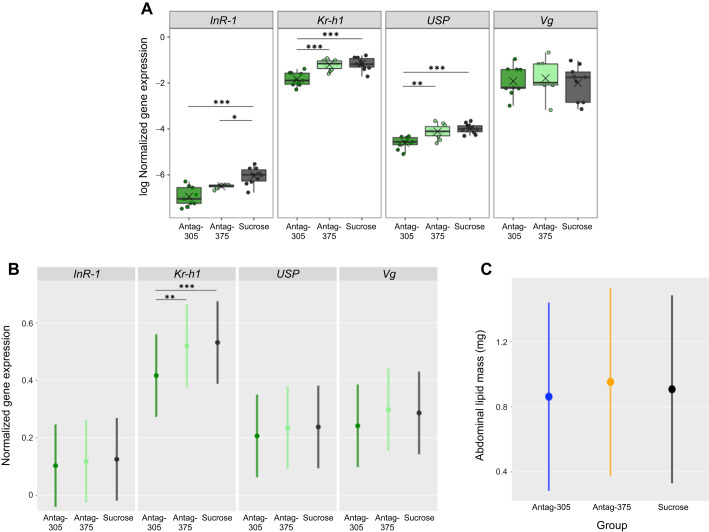
**Antagomir treatment reduced fat body gene expression associated with juvenile hormone (JH), but not insulin/insulin-like signaling, and had no effect on abdominal lipid mass.** (A) Gene expression abundance. ANOVA with Tukey *post hoc*, sample sizes for each gene: *InR-1*, *n*=9 antag-305 treated bees, *n*=7 antag-375 treated bees, and *n*=10 sucrose control bees; *Kr-h1*, *n*=10 bees per group; *USP*, *n*=9 antag-305 treated bees, *n*=10 antag-375 treated bees, and *n*=10 sucrose control bees; *Vg*, *n*=9 antag-305 treated bees, *n*=8 antag-375 treated bees, and *n*=9 sucrose control bees, all bees were from one colony, outliers removed. (B) Repeated downregulation of JH-responsive transcription factor, *Kr-h1*, in the fat body with antag-305. Linear mixed-effects regression model, colony as a random effect, d.f.=486, *n*=42 bees per treatment group per gene, bees were from four colonies. Circles give the means and bars represent 95% confidence intervals. Only significant values are indicated (***P<0.001, **P<0.01, *P<0.05). (C) Abdominal lipid mass. Linear mixed-effects model, colony as a random effect, d.f.=73, *n*=27 antag-305 treated bees, *n*=27 antag-375 treated bees, and *n*=25 sucrose control bees, bees were from four colonies. Points give the means and bars represent 95% confidence intervals. No significant differences observed between treatment groups.

We measured the expression of four genes associated with JH/IIS in the fat body 48 h after antagomir treatment: *insulin-like peptide receptor* (*InR-1*) (Ament et al., 2008), the TF *Krüppel homolog 1* (*Kr-h1*) (Grozinger et al., 2003), the TF *ultraspiracle* (*USP*) (Ament et al., 2012a) and the abdominal lipoprotein *vitellogenin* (*Vg*) (Pinto et al., 2000). In a single trial analysis, the abundance of multiple genes was altered in response to miRNA knockdown relative to the sucrose control group: antag-305 significantly reduced the expression of *InR-1* (a bioinformatically predicted target), *Kr-h1* and *USP* (*P*<0.05, ANOVA with *post hoc* Tukey's test, *n*=9–10 total bees per group, from one colony; Fig. 3A). Additionally, the expression levels of *Kr-h1* and *USP* in the antag-305 group were significantly lower compared with the antag-375 group, indicating that these effects were specific to *miR-305* depletion. Comparison between antag-375 and sucrose control groups showed a significant reduction of *InR-1* (*P*<0.05, ANOVA with *post hoc* Tukey's test, *n*=7–10 total bees per group, from one colony; Fig. 3A). Neither antagomir treatment affected the expression of *Vg*.

We then repeated this analysis with bees from three additional colonies and found that the JH-responsive TF *Kr-h1* was consistently downregulated with antag-305 treatment compared with the sucrose control (*P*<0.001) and the antag-375 group (*P*<0.01, linear mixed-effects regression, *n*=42 total bees per group, from four colonies; Fig. 3B). *InR-1*, the bioinformatically predicted target of *miR-305* (Table S3), was unaffected. Antag-375 treatment did not affect the expression of any of the genes tested. There were no apparent off-target effects.

### No effect of antagomir treatment on abdominal lipid mass

Antagomir-mediated knockdown of *miR-305* and *miR-375* did not induce a change in fat body lipid mass (linear mixed-effects regression, *n*=21–26 total bees per group, from four colonies; Fig. 3C). Mean lipid masses for antag-305, antag-375 and sucrose control groups were 0.862, 0.953 and 0.910 mg, respectively. These quantities are comparable to results from other studies (Ament et al., 2011; Toth and Robinson, 2005).

### JHA and antag-305: JHA reduced lipid levels but antag-305 did not

**Fig. 4.**
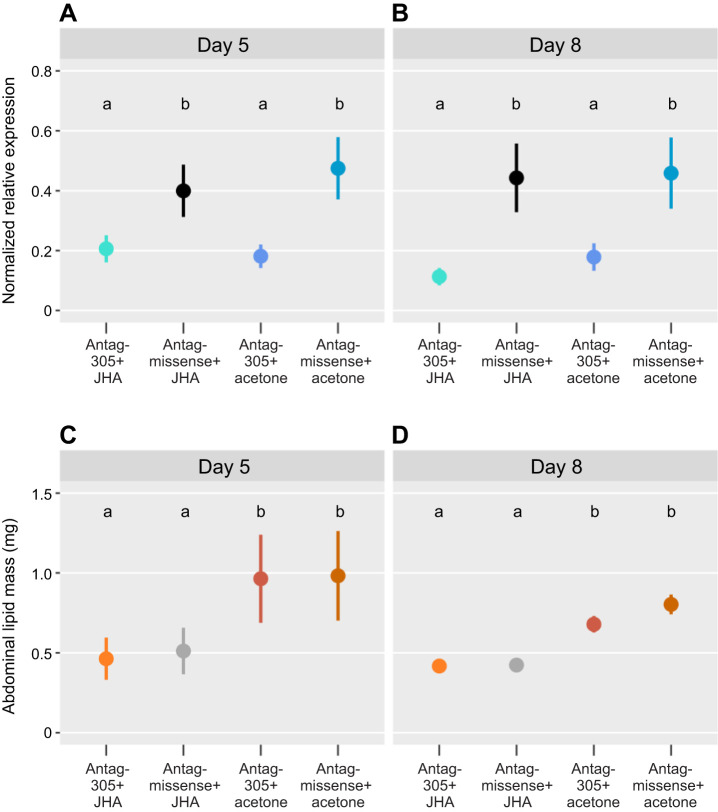
**Juvenile hormone analog (JHA) caused lipid loss and antag-305 reduced *ame-miR-305-5p* expression without synergistic effects.** (A,B) Fat body *ame-miR-305-5p* expression is reduced by antag-305 treatment but unaffected by JHA treatment in (A) 5-day-old bees at day 5 (*P*<0.001, linear mixed-effect regression, colony as a random effect, d.f.=90, *n*=24 antag-305+JHA treated bees, *n*=24 antag-missense+JHA treated bees, *n*=24 antag-305+acetone treated bees, and *n*=23 antag-missense+acetone treated bees, bees were from 2 colonies) and (B) 8-day-old bees at day 8 (*P*<0.0001, d.f.=91, *n*=24 bees per treatment group, bees were from 2 colonies). Circles give the means±s.e.m. (C,D) Abdominal lipid mass is reduced by JHA but not antag-305 treatment in (C) 5-day-old bees at day 5 (*P*<10^−8^, linear mixed-effect regression, colony as a random effect, d.f.=90, *n*=24 antag-305+JHA treated bees, *n*=24 antag-missense+JHA treated bees, *n*=23 antag-305+acetone treated bees, and *n*=24 antag-missense+acetone treated bees, bees were from 2 colonies) and (D) 8-day-old bees at day 8 (*P*<10^−4^, d.f.=91, *n*=24 bees per treatment group, bees were from 2 colonies).

We predicted the combination of antag-305 with JHA would decrease the expression of *ame-miR-305-5p* beyond antag-305 treatment alone. By day 5, miRNA expression in antag-305+JHA-treated bees was reduced by 52% when compared with antag-missense+JHA (*P*<0.0001, linear mixed-effects regression; Fig. 4A). Similarly, antag-305+acetone reduced *ame-miR-305-5p* expression by 62% when compared with antag-missense+acetone (*P*<10^−8^; Fig. 4A). There was no effect of JHA relative to acetone within antag-missense or within antag-305 comparisons, and there were no additive effects from the combined antag-305+JHA treatments on *ame-miR-305-5p* expression.

Results on day 8 were similar to day 5: decreased *ame-miR-305-5p* expression was caused by antag-305 but not JHA treatment (Fig. 4B). Antag-305+JHA reduced *ame-miR-305-5p* expression by 74% compared with antag-missense+JHA (*P*<10^−8^), and antag-305+acetone reduced *ame-miR-305-5p* expression by 61% when compared with antag-missense+acetone (*P*<10^−5^; Fig. 4B). There were no significant effects on *ame-miR-305-5p* expression owing to JHA treatment. Although relative expression levels of *ame-miR-305-5p* between treatment groups remained mostly consistent between day 5 and day 8, the combined antag-305+JHA treatment showed a statistically non-significant trend for lower *ame-miR-305-5p* expression compared with antag-305 alone.

JHA treatment caused lipid loss independent of antag-305. Abdominal lipid mass was reduced by JHA, but not antag-305 treatment, on both day 5 (*P*<10^−8^; Fig. 4C) and day 8 (*P*<10^−6^; Fig. 4D). On day 5, lipid levels for acetone treatments were ∼1 mg, consistent with previous results for bees of this age (Ament et al., 2011; Toth and Robinson, 2005; Toth et al., 2005), whereas levels for JHA-treated bees were ∼0.5 mg, which is the lipid mass typically found in forager bees (Ament et al., 2011). This JHA effect was roughly the same when paired with either antag-305 or missense treatment. Antag-305+JHA reduced lipid mass by 52% compared with acetone control (*P*<10^−9^), and antag-missense+JHA treatment reduced lipid mass by 48% compared with acetone control (*P*<10^−8^; Fig. 4C). On day 8, JHA-treated bees still had lipid levels just below 0.5 mg, whereas acetone-treated bees were ∼0.75 mg. JHA effects were roughly the same when paired with either antag-305 or missense treatment, as on day 5. Antag-305+JHA reduced lipid mass by 39% compared with acetone control (*P*<10^−5^) and antag-missense+JHA reduced lipid mass by 47% compared with acetone control (*P*<10^−8^; Fig. 4D). Thus, there was no additive effect with antag-305+JHA compared with JHA treatment alone at either time point.

### QMP and antag-305: QMP had no effect on fat body *ame-miR-305-5p* expression and neither treatment affected lipid levels

**Fig. 5.**
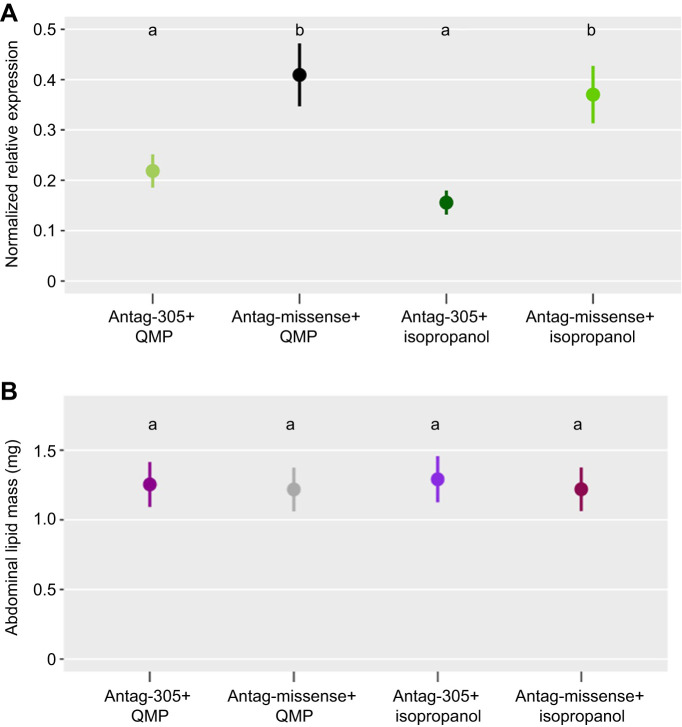
**Queen mandibular pheromone (QMP) had no significant effect on *ame-miR-305-5p* expression and neither treatment factor influenced lipid mass.** (A) Fat body *ame-miR-305-5p* expression was unaffected by QMP compared with isopropanol control treatments in 5-day-old bees (*P*>0.05, linear mixed-effect regression, d.f.=90, *n*=23 antag-305+QMP treated bees, *n*=24 antag-missense+QMP treated bees, *n*=24 antag-305+isopropanol treated bees, *n*=24 antag-missense+isopropanol treated bees, bees were from 2 colonies). Circles give means±s.e.m. (B) Abdominal lipid mass was not influenced by QMP treatment nor antag-305 treatment alone or in any combination at day 5 in 5-day-old bees (*P*>0.05, linear mixed-effect regression, d.f.=175, *n*=45 bees per treatment group, bees were from 2 colonies).

We predicted QMP would increase *ame-miR-305-5p* expression and in combination with antag-305 would result in no change from baseline. Counter to this prediction, antag-305+QMP did not significantly increase *ame-miR-305-5p* expression compared with antag-305 alone (*P*>0.05; Fig. 5A), although mean expression was increased by 37%. There was no difference in *ame-miR-305-5p* expression with antag-missense treatment with or without QMP (*P*>0.05). Overall, QMP did not rescue the effects of antag-305 on fat body *ame-miR-305-5p* expression.

Neither antag-305 nor QMP changed lipid mass in any combination (*P*>0.05; Fig. 5B). Lipid levels across all groups were at approximately 1 mg, consistent with measurements previously obtained for untreated bees of similar age (Ament et al., 2011).

### Antag-305 treatment reduced *ame-miR-305-5p* expression in the fat body but not in the brain

**Fig. 6.**
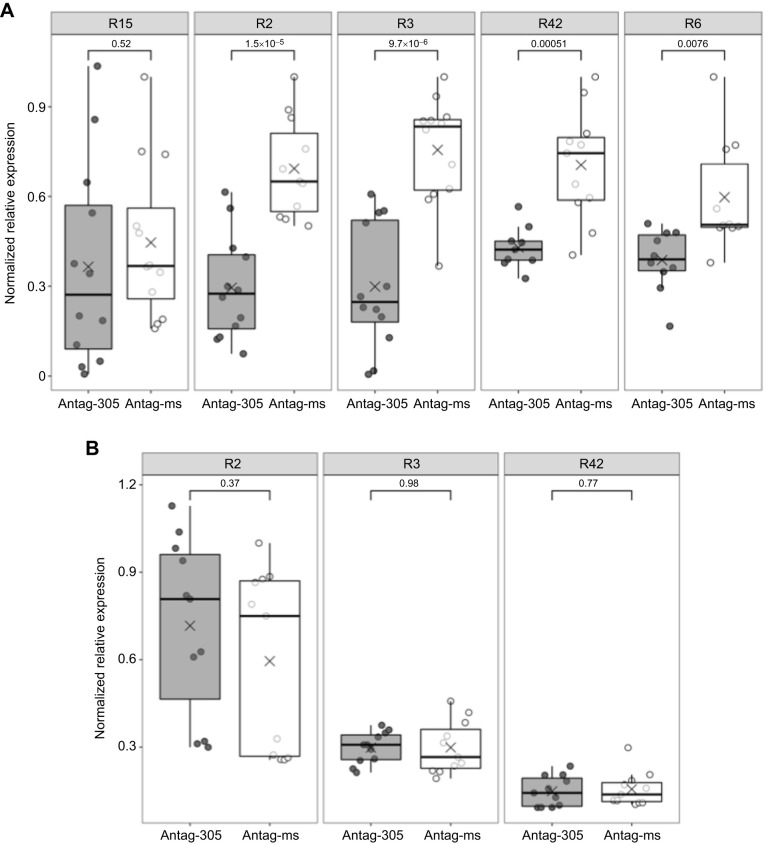
**Antag-305 reduced *ame-miR-305-5p* expression compared with antag-missense in abdominal fat body, but not brain, 48 h after treatment in 5-day-old bees.** (A) Statistically significant knockdown in fat body in four out of five colony trials. Sample size per colony: R15, *n*=11 bees per treatment group; R2, *n*=12 antag-305 treated bees and *n*=11 antag-missense treated bees; R3, *n*=12 bees per treatment group; R42, *n*=9 antag-305 treated bees and *n*=11 antag missense treated bees; R6, *n*=10 antag-305 treated bees and *n*=10 antag-missense, data represented by box plots, Welch's *t*-test, outliers removed. Numbers above brackets represent *P*-values. (B) No effect on *ame-miR-305-5p* expression in the brains of the same bees from three of the same trials. *n*=11 bees per treatment group, Welch's *t*-test. ‘×’ represents the mean and panel labels at the top of each graph (Rx) indicate the colony from which the bees were sourced.

We measured the expression of *ame-miR-305-5p* in the fat body 48 h after antag-305 or antag-missense treatment and found a significant knockdown in miRNA abundance in four out of five trials (*P*<0.01, Welch's *t*-test; Fig. 6A). The average reduction in expression was 48% with a range of 35–61%. These results are consistent with previous antagomir experiments with the sucrose control (Fig. 2) and miRNA knockdown studies in other insects (Cristino et al., 2014; Liu et al., 2017; Lucas et al., 2015c; Yang et al., 2014). The one trial for which there was no difference in expression (Fig. 6A) may have resulted from the genetics of the source colony, indicated by the uncommon production of numerous gynandromorphic bees and roughly double the brood output typically observed from queens inseminated by a single drone (data not shown).

Using the same individuals that were used for the above fat body analysis, we measured brain *ame-miR-305-5p* expression. We only included individuals from three trials that showed a significant knockdown in the fat body. In contrast to fat body results, we found no effects on brain miRNA expression (*P*>0.05, Welch's *t*-test; Fig. 6B).

### Antag-305 treatment caused a large gene expression response in the brain but not in the fat body

**Fig. 7.**
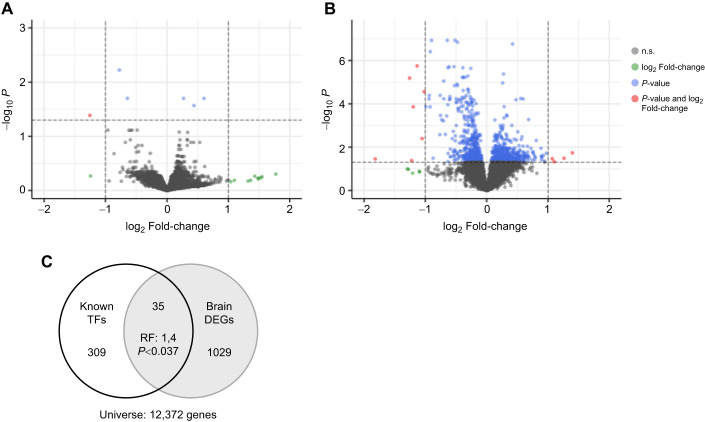
***ame-miR-305-5p* knockdown induced minimal changes in fat body gene expression but large differences in brain gene expression, including numerous transcription factors (TFs).** (A) Abdominal fat body and (B) brain in antag-305 versus antag-missense treated bees (*P*<0.05, FDR). Each point represents an individual gene. Points below zero on the *x*-axis indicate genes with a fold-change increase with antag-305 treatment, and points above zero indicate genes with a fold-change decrease in expression with antag-305 treatment. (C) Differentially expressed TFs in the brain were significantly overrepresented relative to all known honey bee TFs (hypergeometric overlap test). DEGs, differentially expressed genes; RF, representation factor.

Based on miRNA RT-qPCR results above, we anticipated that antagomir treatment would cause effects on gene expression in the fat body, but not the brain. However, we observed the opposite: minimal effects in fat body gene expression but large effects in the brain. In the fat body, only six out of 8844 genes were differentially expressed (FDR<0.05; Fig. 7A). Three genes were upregulated and three were downregulated in the antag-305 group compared with the control antag-missense group (Table S4).

In the brain, by contrast, 1029 out of 9118 genes were differentially expressed as a result of antag-305 treatment (FDR<0.05; Fig. 7B). A total of 497 genes were upregulated and 532 were downregulated (Table S4). DEG results reported here were not affected by Deformed wing virus because samples were screened for high viral load and removed prior to analysis (Table S2)

To better characterize the antag-305 effects on brain gene expression, we performed a TF gene overlap test with annotated honey bee TFs (Wang and Nishida, 2015) and GO enrichment analysis. We predicted high representation of differentially expressed TFs in the brain because two out of six DEGs in the fat body were TFs. This prediction was upheld: 35 out of 309 possible honey bee TFs were affected by antag-305 treatment (Table S5), producing a significant enrichment result (*P*<0.05, representation factor: 1.4, hypergeometric test; Fig. 7C).

GO analysis of genes downregulated by antag-305 in the brain showed enrichment for terms associated with ribosome/structural activity, electron transfer and oxidoreductase activity (Fig. S1A). By contrast, upregulated genes in the brain were significantly enriched for more categories (Fig. S1B), including terms associated with TF and biogenic amine activity. Enrichment of DEGs in categories specific to metabolic, nutrient-sensing or hormonal pathways were not observed.

### *ame-miR-305-5p* perturbation caused differences in brain gene expression related to honey bee division of labor

We explored whether *ame-miR-305-5p* might be involved in the regulation of genes in the brain that are associated with division of labor, but independent of metabolic, nutrient-sensing and hormonal signaling pathways (given the above results on lipid levels). We hypothesized that if *ame-miR-305-5p* regulates genes in the brain that are associated with division of labor, then antag-305 treatment would result in a more forager-like expression pattern. Our prediction was based on detecting lower *ame-miR-305-5p* expression levels in forager fat body and brain compared with nurses (Fig. 1) and the anticipated de-repression of cognate targets associated with behavioral maturation with *ame-miR-305-5p* knockdown.

**Fig. 8.**
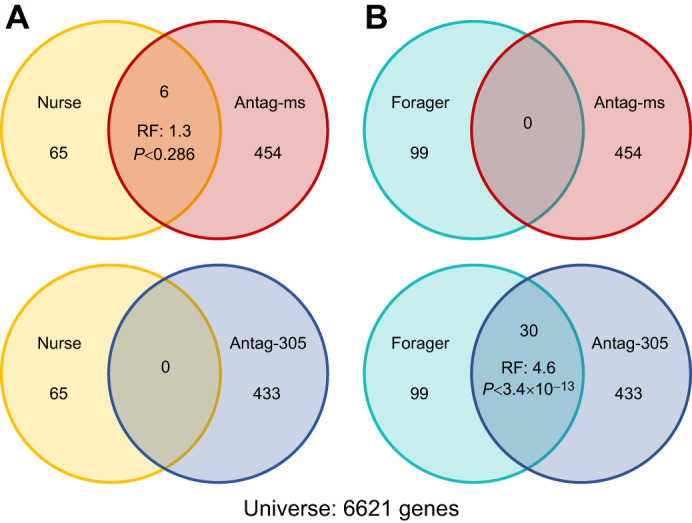
**Antag-305 treatment influenced brain gene expression consistent with patterns previously associated with division of labor (****Alaux et al., 2009****;** **Khamis et al., 2015****)*.*** (A) Nurse-upregulated genes and antag-missense versus antag-305 differentially expressed genes (hypergeometric tests). (B) Forager-upregulated genes and antag-missense versus antag-305 differentially expressed genes (hypergeometric tests).

We first identified 65 concordant nurse-upregulated genes and 99 concordant forager-upregulated genes across two previously published studies (Alaux et al., 2009; Khamis et al., 2015; Traniello et al., 2020). We found that six nurse-upregulated genes overlapped with genes downregulated in the antag-305 group, zero nurse-upregulated genes overlapped with genes upregulated in the antag-305 group, zero forager-upregulated genes overlapped with genes downregulated in the antag-305 group, and 30 forager-upregulated genes overlapped with genes upregulated in the antag-305 group (hypergeometric tests; Fig. 8A,B). All four results were in the predicted direction, but only the fourth overlap result was statistically significant (*P*<10^−6^, representation factor: 4.6; Fig. 8B).

We next determined whether any of the dozens of differentially expressed TFs in our study have been previously implicated in the regulation of division of labor. In a previous study, 15 putative TFs involved in regulating behavioral state were identified as being differentially expressed between nurses and foragers and having DNA binding motifs that were enriched in the promoter regions of other nurse versus forager DEGs: three are predicted regulators of nursing and 12 are regulators of foraging (Khamis et al., 2015). Consistent with our hypothesis, we found that six antag-305-induced differentially expressed TFs were identified as predicted foraging regulators whereas no antag-305-responsive TFs were associated with nursing (Table 2).

**Table 2.**
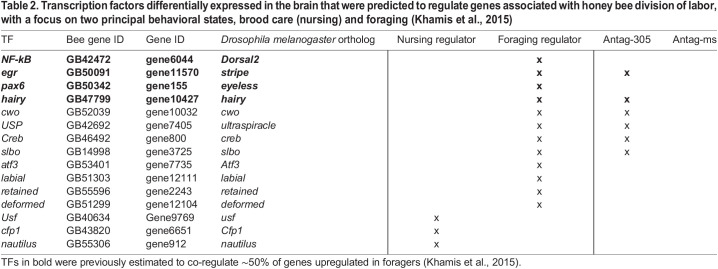
**Transcription factors differentially expressed in the brain that were predicted to regulate genes associated with honey bee division of labor, with a focus on two principal behavioral states, brood care (nursing) and foraging (** **Khamis et al., 2015** **)**

## DISCUSSION

Our two key findings are as follows. First, antagomir-mediated knockdown of *ame-miR-305-5p* did not influence division of labor-related lipid loss in adult honey bee workers alone or in combination with JH or QMP. Second, antagomir-mediated knockdown of *ame-miR-305-5p* caused extensive changes in brain gene expression, particularly for genes previously implicated in division of labor.

In contrast to the brain gene expression results, antagomir-mediated knockdown of *ame-miR-305-5p* caused minimal changes in fat body gene expression which included one JH-associated gene, the JH-responsive TF *Kr-h1*. In honey bees and other social insects, *Kr-h1* is associated with neuroendocrine and behavioral changes, but the function of *Kr-h1* in the fat body has not been described. Based on bioinformatic prediction and results from other insects (Fussnecker and Grozinger, 2008; Gospocic et al., 2021; Grozinger and Robinson, 2007), we had expected to see transcriptomic evidence for *ame-miR-305-5p* regulation of IIS, which has been implicated in the regulation of division of labor (Ament et al., 2008; Hamilton et al., 2017). JH and IIS pathways have been shown to interact in honey bees (Corona et al., 2007); however, no effects were detected for other JH and IIS associated genes.

Consistent with the limited effects of *ame-miR-305-5p* fat body knockdown on fat body gene expression, there also was no effect on lipid levels. By contrast, there was a strong reduction of abdominal lipid mass with JHA treatment, an effect that has long been assumed but never directly measured (Ament et al., 2012a). That this occurred several days earlier than the onset of precious foraging with the same treatment (Robinson, 1987; Sullivan et al., 2000) is consistent with previous results showing that changes in lipid level precede, and are causal to, changes in behavior (Toth and Robinson, 2005; Toth et al., 2005).

We predicted QMP would increase lipid mass and upregulate *ame-miR-305-5p* expression whereas *ame-miR-305-5p* knockdown in the fat body would act in opposition to both. Our results showed no statistical effect of either treatment on lipid level or *ame-miR-305-5p* expression; however, we detected a 37% increase with QMP that may indicate an antagonistic interaction of QMP and antag-305 on the fat body expression of *ame-miR-305-5p*. This would suggest that QMP increases *ame-miR-305-5p* expression as originally hypothesized, which is consistent with the presence of high *ame-miR-305-5p* abundance in nurse bees, as they are exposed to relatively higher levels of QMP than other groups of workers in honey bee colonies. Whether the decline in *ame-miR-305-5p* with behavioral maturation is associated with the decline in QMP efficacy in older bees (Vergoz et al., 2009) is unknown. The lack of increase in lipid levels with QMP is contrary to previous studies (Ament et al., 2011; Fischer and Grozinger, 2008).

Antagomir-mediated knockdown of *ame-miR-305-5p* in abdominal fat body caused extensive changes in brain gene expression, but not in fat body, particularly genes expressed in the brain previously implicated in division of labor. We do not have a mechanistic explanation for this but provide two speculative possibilities below.

One possible explanation for the finding of extensive changes in gene expression in the brain owing to treatment administered to the fat body is signaling from the fat body. This speculation is based on the finding that fat body knockdown affected fat body expression of the TFs *hairy* and *E(spl)m7*; both *hairy* and *E(spl)m7* are conserved bHLH repressors, which act as downstream effectors of Notch in insects (Dearden, 2015) and vertebrates (Gibb et al., 2010; Kawamura et al., 2005). In honey bees, Notch is involved in the regulation of reproductive division of labor (Duncan et al., 2016), and the Notch pathway, along with insulin signaling, has been shown to be coordinated by *miR-305* in *D. melanogaster* gut (Foronda et al., 2014). Whether *ame-miR-305-5p* interacts with Notch to influence bHLH activity and behavioral maturation, or whether Notch is involved at all, is still unknown. *E(spl)m7* has not previously been shown to be involved in honey bee behavioral maturation.

*Hairy*, by contrast, has been implicated in honey bee behavioral maturation, specifically the hive bee to forager transition (Khamis et al., 2015; Sinha et al., 2006). It is upregulated in forager brains compared with nurses (Khamis et al., 2015), and the promoter regions of hundreds of forager-associated genes are enriched for DNA binding motifs of *hairy* (Khamis et al., 2015; Sinha et al., 2006). Our results show that *hairy* is upregulated in both the fat body and brain, suggesting it may be an important *ame-miR-305-5p* regulated gene. Whether *ame-miR-305-5p* represses *hairy*, or any other cognate target, via direct interactions in both tissues or just one, or is an indirect target is still unknown and cannot be differentiated with the methods used to generate results here.

The hypothesis of signaling between fat body and brain should be tested empirically, but extensive connections between fat body and brain gene expression networks in honey bees have been predicted from computational analyses (Ament et al., 2012a). In addition, other studies have shown that extensive changes in brain gene expression can be elicited by treatments administered to other peripheral tissues, including the fat body (Ament et al., 2012a; Wheeler et al., 2013). There also is evidence of similar effects of the ovaries, which are thought to be involved in the regulation and evolution of social behavior (Graham et al., 2011; Wang et al., 2012). To our knowledge, this is the first time a miRNA has been implicated in cross-tissue coordination of gene expression.

A second possible explanation for the finding of extensive changes in gene expression in the brain owing to treatment administered to the fat body comes from findings in vertebrates, where TFs have been proposed to be secreted and act as signaling molecules that play a role in intercellular transfer and non-cell autonomous activity (Di Nardo et al., 2018; Lee et al., 2019), which can influence behavior. For example, TF OTX-2 in the mouse is secreted from the choroid plexus in the brain into the cerebrospinal fluid, where it is then taken up by inhibitory interneurons to regulate anxiety-like behavior (Vincent et al., 2021).

As noted above, it is surprising that a ∼50% decrease in fat body *ame-miR-305-5p* expression was sufficient to cause differences in the expression of over 1000 genes in the brain, especially given that brain *ame-miR-305-5p* expression was unaffected by the antag-305 treatment. Transcriptomic effects included genes related to biogenic amines, including octopamine and serotonin, and behavioral maturation-associated TFs. It would be interesting to determine whether the potent effect of this abdominal treatment on the brain is due to direct or indirect interactions; recently, it was shown that the blood–brain barrier is involved in the regulation of JH-related division of labor in *Camponotus floridanus* carpenter ants (Ju et al., 2023).

Several biogenic amines have been shown to exert causal effects on behavioral maturation and other aspects of division of labor (Barron et al., 2010). Few miRNAs have been associated with biogenic amine activity (Shen et al., 2020), and no effects have been reported for *miR-305* until now. TFs have been studied extensively in the context of honey bee division of labor; some have been shown to have causal effects on behavior (Ament et al., 2012a) and effects that allow brain gene regulatory networks to ‘rewire’ to support different behavioral states (Chandrasekaran et al., 2011; Hamilton et al., 2019; Khamis et al., 2015). This includes *egr*, *clockwork orange*, *hairy*, *ultraspiracle*, *Creb* and *slbo*, all of which showed effects of *miR-305* knockdown. Given that there were no effects on gene expression related to JH/IIS, two pathways known to also be involved in the regulation of division of labor, our findings are consistent with observations that division of labor is influenced by multiple, independent pathways (Ament et al., 2012b; Whitfield et al., 2006).

The extensive changes in brain gene expression caused by knockdown of *ame-miR-305-5p* shifted brain gene expression pattern to being more forager-like. This is consistent with the observation that *ame-miR-305-5p* expression is lower in foragers bees than nurse bees, suggesting that antag-305 de-repressed gene expression associated with behavioral maturation. Different factors have also been found to shift brain gene expression to a forager-like state, including viral infection (Traniello et al., 2020), JH (Wheeler et al., 2015; Whitfield et al., 2006) and manganese (Whitfield et al., 2006). This study is only the second to use manipulations of a miRNA to gain insights into the regulation of division of labor. Liu et al. (2017) focused on division of labor-related sensory physiology, and the present study focused on division of labor-related brain and fat body gene expression.

In summary, this study adds to our understanding of the mechanisms governing division of labor in honey bee colonies. We have shown causal effects of a specific miRNA on brain gene expression, affecting molecular pathways known to be involved in some, but not all, aspects of division of labor. Lack of effects of this miRNA on the regulation of abdominal lipids highlights the fact that although division of labor involves coordinated changes in physiology and behavior, they are not all regulated in the same way.

## Supplementary Material

10.1242/jexbio.246785_sup1 Supplementary information

Table S3. Bioinformatic screening for miRNAs that regulate juvenile hormone (JH) and insulin/insulin-like signaling (IIS). A) Results from RNAhybrid and B) PITA, run with recommended settings.

Table S4. Differentially expressed genes (DEGs) in abdominal fat body and brain in response to ame-miR-305-5p knockdown in the fat body. Colony as a blocking factor, FDR < 0.05. Includes logFC, gene identifiers, gene descriptions, and D. melanogaster orthologs.

Table S5. Differentally expressed transcription factors in the honey bee brain with ame-miR-305-5p knockdown. p < 0.05, hypergeometric test.
