# Supplementary material for: Effects of microRNA-305 knockdown on brain gene expression associated with division of labor in honey bee colonies (Apis mellifera)
Source: J Exp Biol. 2024 Apr 30;227(8):jeb246785. doi: 10.1242/jeb.246785 (PMC11112348; doi:10.1242/jeb.246785)
Supplement: Supplementary information [file jexbio-227-246785-s1.pdf]

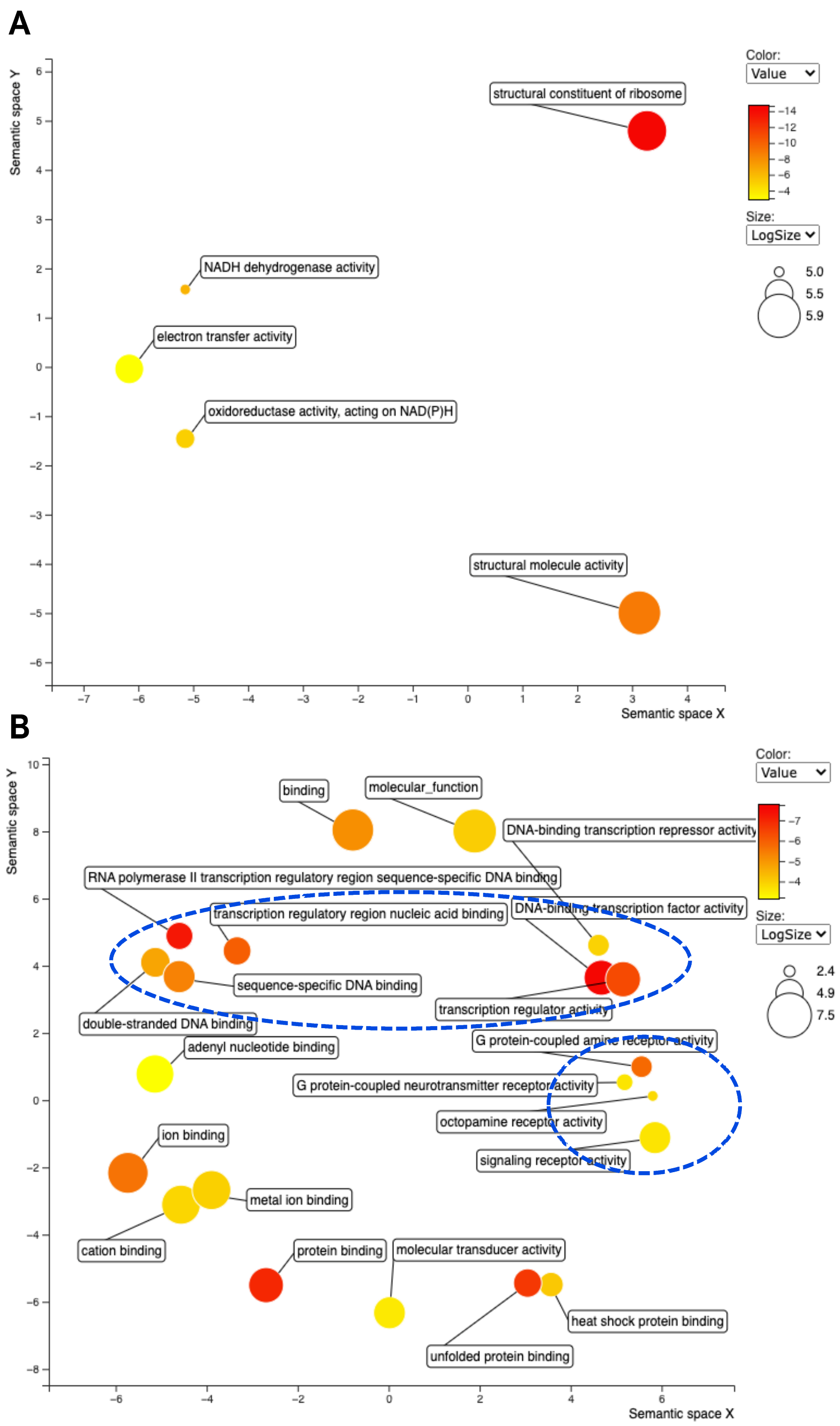

Fig. S1. Molecular function terms enriched from Gene Ontology analysis of antag-305 effects on brain gene expression for A) genes downregulated by antag-305 treatment and B) genes upregulated by antag-305 treatment. Dotted circles highlight two clusters of interest related to transcription factor activity (top) and biogenic amine activity (bottom).

Journal of Experimental Biology • Supplementary information

Table S1. Nucleotide sequences for RT-qPCR primers and antagomir constructs.

**miRNA qPCR**	**Sequence (5' – 3')**
cel-miR-39-3p spike-in	Proprietary (Qiagen, Hilden, Germany)
cel-miR-39-3p forward primer	Proprietary (Qiagen, Hilden, Germany)
Universal reverse primer*	GAATCGAGCACCAGTTACGC
ame-miR-210-3p forward primer**	TTGTGCGTGTGACAGCGGCTA
ame-miR-278-3p forward primer	TCGGTGGGACTTTCGTCCGTTT
ame-miR-305-5p forward primer	ATTGTACTTCATCAGGTGCTCTG
ame-miR-375-3p forward primer	TTTGTTCGTTCGGCTCGAGTTA
ame-miR-6043-3p forward primer	ATGGTGACCGTGATCTATTCCA
ame-miR-6056-5p forward primer	GAGGGACGAGGAGGGAGGTT
ame-miR-9873-3p forward primer	GATTATTTGCTGCGGCTTTGGA
**Antagomir construct**	**Sequence (5' – 3')**
Antagomir-210	UAGCCGCUGUCACACGCACAA
Antagomir-305	CAGAGCACCUGAUGAAGUACAAU
Antagomir-375	UAACUCGAGCCGAACGAACAAA
Antagomir-6056	AACCUCCCUCCUCGUCCCUC
Antagomir-9873	UCCAAAGCCGCAGCAAAUAAUC
**mRNA qPCR**	**Sequence (5' – 3')**
InR-1 forward primer	ACGGGATGGCCTACTTGGAG
InR-1 reverse primer	GGAAACCATGCAATTCCTCG
Kr-h1 forward primer	GCACTGGCAGTGACAAGGAA
Kr-h1 reverse primer	CGTGGAGTGTTATCGTAAGTAGCAA
USP forward primer	AGGGTTATTGCCGCGTAGCT
USP reverse primer	CGGGCAGGCGTAGAAGTAATT
Vg forward primer	AGTTCCGACCGACGACG
Vg reverse primer	TTCCCTCCCACGGAGTCC
Rp49 forward primer	GGGACAATATTTGATGCCCAAT
Rp49 reverse primer	CTTGACATTATGTACCAAAAC TTTTCT
S8 forward primer	TGAGTGTCTGCTATGGATTGCAA
S8 reverse primer	TCGCGGCTCGTGGTAAA
GapDH forward primer	ACTGGTATGGCCTTCCGTGTAC
GapDH reverse primer	TGCCAAGTCTAACTGTTAAGTCAACA

*Obtained from Bryant et al,.2010
**Obtained from Cristino et al., 2014

Table S2. Samples removed from RNA-Seq analysis for Deformed wing virus (DWV) contamination.

**GSE200602 ID**	**Sample ID**	**Tissue**	**Colony**	**Treatment**	**Bee**
GSM6038718	R3_ams_FB21	Abdominal fat body	R3	antag-ms	21
GSM6038757	R42_ams_FB15	Abdominal fat body	R42	antag-ms	15
GSM6038669	R2_ams_FB23	Abdominal fat body	R2	antag-ms	23
GSM6038672	R2_ams_FB27	Abdominal fat body	R2	antag-ms	27
GSM6038706	R3_ams_B21	Brain	R3	antag-ms	21
GSM6038746	R42_ams_B15	Brain	R42	antag-ms	15

Journal of Experimental Biology • Supplementary information

Table S3. Bioinformatic screening for miRNAs that regulate juvenile hormone (JH) and insulin/insulin-like signaling (IIS). A) Results from RNAhybrid and B) PITA, run with recommended settings.

Available for download at
https://journals.biologists.com/jeb/article-lookup/doi/10.1242/jeb.246785#supplementary-data

Table S4. Differentially expressed genes (DEGs) in abdominal fat body and brain in response to ame-miR-305-5p knockdown in the fat body. Colony as a blocking factor, FDR < 0.05. Includes logFC, gene identifiers, gene descriptions, and D. melanogaster orthologs.

Available for download at
https://journals.biologists.com/jeb/article-lookup/doi/10.1242/jeb.246785#supplementary-data

Table S5. Differentally expressed transcription factors in the honey bee brain with ame-miR-305-5p knockdown. $p < 0.05$, hypergeometric test.

Available for download at
https://journals.biologists.com/jeb/article-lookup/doi/10.1242/jeb.246785#supplementary-data
